# Mosquito-borne infectious diseases in northeastern Italy: analysis of social factors influencing risk perception among the population

**DOI:** 10.3389/fpubh.2025.1741038

**Published:** 2026-01-06

**Authors:** Giulia Mascarello, Anna Pinto, Stefania Crovato, Marco Zago, Francesco Gradoni, Fabrizio Montarsi

**Affiliations:** 1 Communication Unit, Istituto Zooprofilattico Sperimentale delle Venezie, Legnaro, Padua, Italy; 2 Laboratory of Medical Entomology and Vector-borne Diseases, Istituto Zooprofilattico Sperimentale delle Venezie, Legnaro, Padua, Italy

**Keywords:** attitude, citizen practice, healthprotection, one health, public health, risk perception and knowledge, vector control, vector-borne diseases

## Abstract

**Introduction:**

Northeastern Italy has the highest reported circulation of mosquito-borne diseases (MBDs) in Europe, due to the considerable diversity of species and the abundance of mosquitoes in the area. This study aims to investigate knowledge, attitude and practice (KAP) indicators related to mosquitoes and MBDs and to identify the key determinants influencing risk perception among the population of northeastern Italy, including the role of social norms and media exposure.

**Methods:**

Data were collected through a structured questionnaire administered using a mixed-mode approach, combining online and telephone interviews. The target population of the survey consisted of people aged 18 years and older, living in the Veneto and Friuli Venezia Giulia Regions.

**Results:**

Respondents showed strong awareness of mosquito breeding sites and were conscious of the existence of invasive species. However, there were some misconceptions about mosquito biology and limited knowledge of locally present MBDs. The population reported engaging in a wide range of protective behaviors, including the use of repellents and physical barriers such as mosquito nets. Respondents with higher risk perception were more likely to adopt protective behaviors, but were less knowledgeable about mosquito biology and ecology. In addition, sociodemographic variables, descriptive social norms, and exposure to media reports proved to significantly influence perceived risk.

**Discussion:**

Understanding public perceptions and attitudes toward mosquitoes is an essential part in the design of risk communication strategies and in planning citizen engagement in prevention measures to protect public health. To reduce the health risks associated with mosquito bites, an interdisciplinary, multi-stakeholder approach is recommended, combining epidemiological surveillance, education, communication, and community mobilization. Based on the findings, several recommendations are advanced to improve mosquito control efforts and public health communication strategies.

## 1 Introduction

Globalization, intensified trade and the movement of people and goods have facilitated the spread and establishment of vectors and their pathogens (1). Furthermore, climate change is creating favorable environmental conditions for mosquito population growth and the spread of mosquito-borne diseases (MBDs) in non-endemic regions such as Europe (2).

In Italy around 65 native mosquito species are present, to which invasive exotic species have been added (3). *Aedes albopictus* (the tiger mosquito), originally from south-east Asia, has spread rapidly since the 1990s and is now established throughout Italy. Other invasive species have been introduced into Italy more recently such as *Aedes koreicus* (the Korean mosquito), first detected in 2011 in the province of Belluno, Veneto Region (4), and *Aedes japonicus* (the Asian bush mosquito), reported in 2015 in the province of Udine, Friuli Venezia Giulia Region (5). *Aedes* species are of particular concern because they are vectors of more than 100 human pathogens, among which dengue, Zika and chikungunya are the most significant (6).

In addition, some mosquito species of the genus *Anopheles*, belonging to the *Maculipennis* complex, are still present in Italy (7). This complex includes species that are potential vectors of malaria, a disease eradicated in Italy since the 1970s but which could re-emerge, considering their local abundance in some parts of Italy.

The North East is among the areas with the greatest species diversity and abundance in Italy (8). As a result, the region also shows some of the highest levels of MBD circulation in Europe (2). West Nile virus (WNV) has been circulating since 2008 (9), while many imported cases of chikungunya, Zika, and dengue (contracted by travelers in endemic regions) are reported each year (2). Notably, the first autochthonous outbreak of dengue in Italy was recorded in the Veneto Region (10).

National and local health authorities are increasingly involved in the implementation of surveillance systems targeting the presence of mosquitoes and MBDs, alongside vector control interventions aimed at curbing mosquito proliferation. Effective vector management at the local level requires coordinated efforts, including citizen engagement, since mosquito control necessitates integrated actions and the commitment of all stakeholders operating within the territory (11–13). Citizens play a pivotal role in reducing mosquito habitats and densities (14). Public awareness and educational campaigns promoting personal protection and environmental source reduction are core components of the Integrated Vector Management (IVM) strategy recommended by the World Health Organization (15). In recent years, the adoption of IVM principles has led to the development and testing of citizen science initiatives, whereby the public is directly involved in passive mosquito surveillance through digital platforms such as Mosquito Alert, iNaturalist, Mosquito Habitat Mapper, and GLOBE Observer. These initiatives enhance public engagement and leverage both scientific and local experiential knowledge (16–18).

The prevention of MBDs requires a comprehensive understanding of the ecological, behavioral, and social determinants that influence mosquito proliferation and human exposure. While entomological surveillance remains essential for identifying invasive mosquito species and monitoring the circulation of pathogens of medical and veterinary significance, it is also important to examine the sociocognitive factors that shape preventive practices among the population. These practices include voluntary actions aimed at reducing mosquito bites, such as using repellents, wearing protective clothing, and eliminating breeding sites. However, the decision to engage in protective behaviors results from a complex interplay of factors, including socioeconomic determinants, awareness, knowledge, and perceived risk (19, 20). In this framework assessing public Knowledge, Attitudes, and Practices (KAP) regarding mosquitoes and MBDs is essential to designing targeted, effective risk communication strategies.

Additionally, individual behaviors can be shaped by perceptions of others’ opinions and practices, known as descriptive social norms. Numerous studies have demonstrated the influence of meaningful social referents, such as family members and neighbors (21–23). Moreover, personal experiences and perceived exposure to risk also play a significant role in the adoption of Personal Protective Behaviors (PPBs). Previous research has shown that media represent a major source of information for individuals who lack direct experience with vector-borne diseases (24).

This study aims to investigate KAP indicators related to mosquitoes and MBDs and to identify key determinants influencing risk perception among the population of northeastern Italy, including social norms and media information exposure.

## 2 Materials and methods

A structured questionnaire (25) was developed based on the research team’s expertise and the existing literature (26–29). The questionnaire consisted of 22 questions exploring the following topics: attitudes (perceptions of mosquito-related risks and personal experiences); knowledge (of breeding sites, mosquito ecology/biology, and MBD); protective and control practices (adoption of PPBs against mosquito bites, mosquito control actions, and perceived responsibility for mosquito control); descriptive social norms; exposure to media information; and sociodemographic data.

Experts in entomology and vector-borne disease control and prevention contributed to the development of all the section of the questionnaire, both identifying key health issues to investigate and ensuring the accuracy of the contents and of the questions and answer wording.

Table 1 presents the questions and corresponding response formats for each question. The questionnaire was pre-tested with 13 citizens prior to administration to identify any unclear or ambiguous questions. Following the pre-tests, the questionnaire was slightly modified and shortened. Some questions and response options were removed and some scales were modified (from Likert to dichotomous) to simplify the completion process. To avoid response order bias in questions with multiple items, the items were rotated during the administration of the questionnaire (30).

**Table 1:** Items used and the response format for each question.

Items	Response format
Attitudes	
In the municipality where you live, do you think the presence of mosquitoes over the past 2–3 years has become…	Scale: very prevalent/quite prevalent/not very prevalent/not prevalent at all/I do not know
In the municipality where you live, do you think the presence of mosquitoes over the past 2–3 years has…	Scale: increased/decreased/remained the same
In your opinion, why have mosquitoes increased in recent years?	Open answer
To what extent do you agree with the following statements? I think mosquitoes are very annoying. Mosquitoes are a serious threat to my health	Likert scale 1–7, where 1 = not agree at all, 7 = agree a lot
How concerned are you about the possibility of contracting a serious disease from mosquito bites in your province? And in Italy?	Likert scale 1–7, where 1 = Not at all concerned, 7 = Very concerned
Knowledge	
*Mosquito reproductive sites*: Based on your personal knowledge, can you identify which environments favor mosquito proliferation? List of environments	Yes/No/I do not know
*Mosquito ecology/biology*: Based on your knowledge, indicate whether each statement is true or false. Items on mosquito ecology (see Table 5)	True/false
*Mosquito species*: In your opinion, in your area there are… Only native mosquitoes; Only mosquitoes from other countries; Both native and exotic mosquitoes	Single choice answer
*Mosquito-transmitted diseases*: Mosquito bites can transmit some agents of diseases. Can you say whether the following diseases have been reported in your area (province) in recent years? List of diseases (see Figure 2)	Yes/No/I do not know
Protection and control practices	
Personally, which methods do you usually use to protect yourself from mosquitoes? List of control measures/methods (see Table 2)	Multiple choice answer
In your opinion, to what extent should the following figures actively participate in mosquito control efforts by taking specific prevention actions?	Scale: not at all/slightly/fairly/very much/I do not know
Descriptive social norms	
To what extent do you agree with the following statements? Most people living in my area believe that mosquitoes are very annoying; Most people living in my area believe that mosquitoes are a serious threat to the community	Likert scale 1–7, where 1 = not agree at all, 7 = agree a lot
Media information exposure	
Have you recently come across news about mosquitoes in newspapers or on television?	Yes/no

Data were collected between September 11 and 22, 2020, using a mixed-mode approach combining Computer-Assisted Web Interviewing (CAWI) and Computer-Assisted Telephone Interviewing (CATI). This methodology was adopted to ensure adequate coverage of both younger and older segments of the population. Participant recruitment and questionnaire administration were supported by the research firm Demetra Opinioni.net S.r.l.^1^

The target population of the survey consisted of residents aged 18 years and older living in northeastern Italy (Veneto and Friuli Venezia Giulia Regions), who either had a landline telephone or were registered in the Opinioni.net online panel. A representative sample was drawn from this population, stratified by gender, age group, and province of residence to reflect the demographic distribution of the reference population. A total of 1,001 individuals completed the questionnaire, including 600 residents from the Veneto and 401 from Friuli Venezia Giulia. Equal quotas of respondents were maintained in the two Regions for CAWI and CATI methods (Veneto: 300 CATI interviews, 300 CAWI interviews; Friuli Venezia Giulia: 201 CATI interviews, 200 CAWI interviews).

Informed consent for participation was obtained through an opt-in checkbox at the beginning of the questionnaire, in compliance with privacy regulations. All data were collected anonymously and processed in accordance with the General Data Protection Regulation (EU) 2016/679.

### 2.1 Statistical analysis

Univariate statistics (percentages, means, or medians in the case of highly skewed distributions) were calculated for the variables, according to type.

Pearson’s correlation coefficient was computed between level of agreement with the statement, “Mosquitoes are a serious threat to my health” and perceived level of concern about potentially contracting a serious disease from mosquito bites in the respondent’s province of residence. The individual risk perception index (RP index) was then created by calculating the mean value of these two variables. The two variables refer to perceived susceptibility, i.e., the perception of being at risk for the condition, and the perception of risk severity, i.e., the seriousness of the condition. Severity and susceptibility dimensions were selected to compose the RP index because some of the main behavior change models, such as the Health Belief Model and Protection Motivation Theory, identify these constructs as the components of the threat appraisal process, which is a key element in influencing behaviors (31).

Variation in the RP index across sociodemographic variables was investigated using the independent samples *t*-test for two independent groups, and one-way analysis of variance (ANOVA) with Gabriel’s *post-hoc* correction for comparisons of more than two groups.

The “protective behaviors” variable was built by summing 14 dichotomous variables that captured the adoption of protective behaviors. For the protective behaviors variable, the missing data correspond to individuals who indicated that they do not use any mosquito-protection system. Their answers were therefore coded as individual “I do not use it” responses for each protective behavior.

A knowledge variable was created by summing the correct answers to 6 questions (true/false/I do not know) about mosquito ecology and biology. For knowledge variable “do not know” responses were recoded together with the incorrect ones as “other” responses.

After verifying the linear relationship between variables, in the absence of outliers and given the large sample size. Pearson’s correlation coefficient was calculated between the RP index and continuous variables, such as respondents’ knowledge, and protective behaviors.

Cronbach’s alpha coefficient was calculated to assess the internal consistency of the multi-item variables measured on a quantitative scale.

Data were analyzed using Statistical Package for Social Sciences (SPSS) software (version 25.0) for Windows (SPSS Inc., Chicago, Illinois). The level of statistical significance was set at 5% (*α* = 0.05).

## 3 Results

### 3.1 Characteristics of the sample

The majority of the sample, consisting of 1,001 respondents, was female (54.6%), aged between 45 and 64 years (36.6%), had children (60.8%), had a high school diploma (37.9%), and defined themselves as employed (47.7%). Table 2 provides a detailed overview of the respondents’ characteristics. When asked to describe the area and setting in which they lived, most respondents (72.4%) reported residing in a low-lying area, while 13.1% lived in hilly areas, 6.7% in mountainous areas, and 7.8% along the coast or on islands. Furthermore, 63.3% reported living in an urban environment and 36.7% in a rural setting. Approximately 81% of respondents declared to have never traveled to tropical countries.

**Table 2:** Characteristics of the sample (%, *n* = 1,001).

Characteristics	%
Gender	
Female	54.6
Male	45.4
Age (in groups)	
18–29	13.1
30–44	22.4
45–64	36.6
65 and over	27.9
Presence of children in the family	
Yes	60.8
No	39.2
Academic qualification	
Up to elementary school certificate	8.9
Middle school diploma	17.4
High school diploma	37.9
Vocational qualification	8.1
University degree	24.3
Postgraduate specialization	3.4
Employment status	
Student	7.8
Employed	47.7
Unemployed	6.6
Homemaker	7.9
Retired	30.0

### 3.2 Attitudes and perceptions

Most respondents (82.8%) perceived a high presence of mosquitoes in their area. When comparing the current situation to the past 2–3 years, 44.5% perceived an increase in the presence of mosquitoes, 45.6% reported perceiving no change, and 10% perceived a decrease. Those reporting an increase attributed it primarily to environmental factors, such as climate change and the presence of standing water, and secondarily to human behaviors, including the lack of disinfestation efforts or insufficient preventive measures.

In general, respondents expressed a relatively high level of agreement with the statement, “I think mosquitoes are very annoying” (median = 7; IQR = 17), while there was less agreement with the statement, “Mosquitoes are a serious threat to my health” (mean = 3.95; SD = 1.948).

Respondents were less concerned about contracting a serious MBD in their own province compared to contracting one elsewhere in Italy (Table 3).

**Table 3:** Level of concern about of contracting a serious disease from mosquito bites in own province of residence or elsewhere in Italy (Likert scale 1–7, where 1 = not at all concerned, 7 = very concerned; *n* = 1,001).

Items	Mean	SD	*t*-test	*p*-value
In the province of residence	3.16	1.941	13.216	0.000
Elsewhere in Italy	3.69	1.910		

Cronbach’s alpha coefficient indicated satisfactory internal consistency for the two items presented in Table 3 (*α* = 0.876).

Findings showed a strong positive correlation between the statement, “Mosquitoes are a serious threat to my health” and perceived level of concern about contracting a serious disease from mosquito bites in own province of residence (Pearson’s correlation coefficient *r* = 0.528, *p*-value<0.001).

### 3.3 Knowledge

#### 3.3.1 Knowledge of mosquito reproductive sites

Respondents identified natural or artificial places with standing water, such as ponds and swamps (97.1%), plant saucers, flower pots, watering cans (92.1%), rainwater collection containers (89.5%), and catch basins (83.9%) as being environments that promote mosquito breeding.

#### 3.3.2 Knowledge of mosquito biology/ecology

Scientific knowledge about mosquito biology and ecology was assessed using true/false items. The statement with the highest proportion of incorrect responses (71%) was “Mosquitoes feed only on blood.” Conversely, the statement with the highest proportion of correct responses (76.5%) was, “Mosquitoes bite only people and not animals” (Table 4).

**Table 4:** Distribution of correct and incorrect responses to questions on knowledge of mosquito biology/ ecology (%; *n* = 1,001).

Items	Correct response	Incorrect response
Only female mosquitoes bite (True)	52.9	47.1
A mosquito bite can, in some cases, transmit pathogens that cause diseases (True)	**83.7**	16.3
In Italy, the risk of contracting serious infections from mosquito bites is rare (True)	58.7	41.3
Mosquitoes feed only on blood (False)	29	**71**
Mosquitoes bite only people and not animals (False)	**76.5**	23.5
Different mosquito species can transmit different diseases (True)	**75.4**	24.6

#### 3.3.3 Knowledge of mosquito species

**Figure 1:**
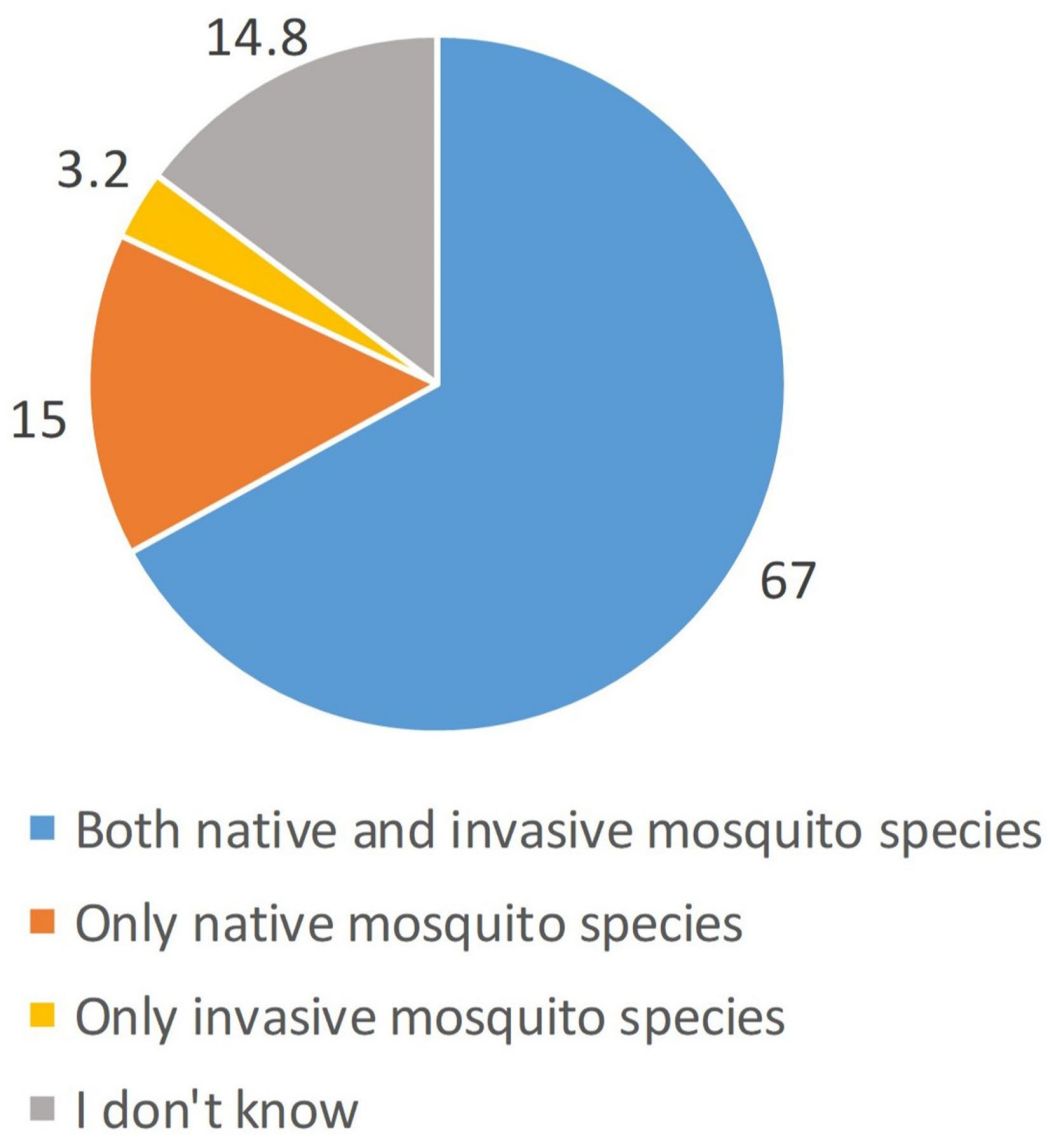
Mosquito species that respondents believed to be present in their area of residence (%, *n* = 1,001).

Most respondents (67%) believed that both native and invasive mosquito species were present in their area of residence (Figure 1).

#### 3.3.4 Knowledge of mosquito-transmitted diseases

**Figure 2:**
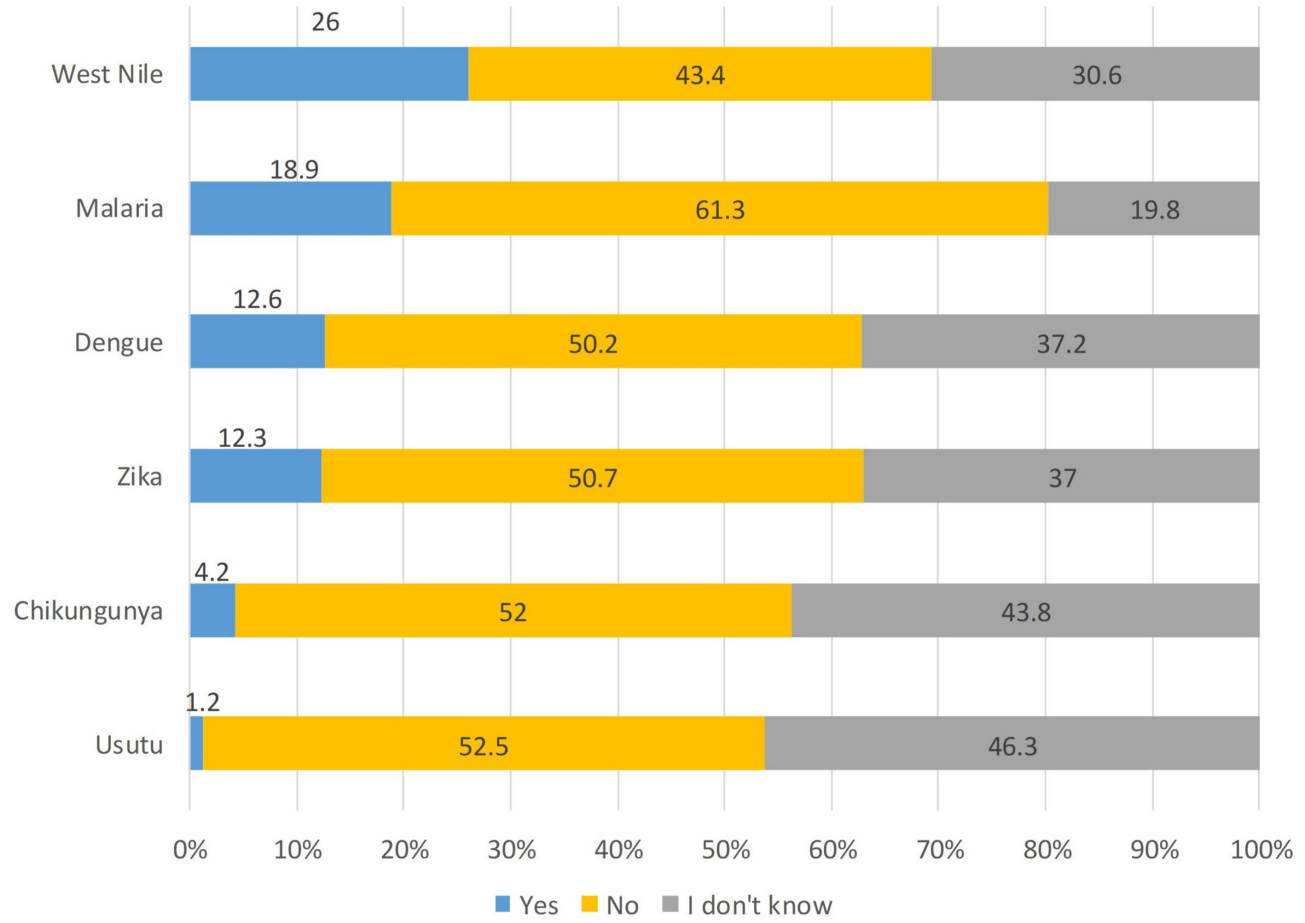
Diseases that respondents believed had been identified in their area of residence in recent years (%, *n* = 1,001).

When asked to identify which MBDs had been reported in their area of residence, respondents most frequently mentioned West Nile disease (WND), followed by malaria and dengue. A high proportion of “I do not know” responses was observed across all listed diseases (Figure 2).

### 3.4 Personal protective behaviors

The most commonly reported PPBs against mosquito bites included the use of skin-applied repellents (62.6%), followed by environmental repellents such as mosquito coils, electric diffusers (60.1%), and mosquito nets (53.5%).

In addition to PPBs, respondents reported taking actions to reduce the presence of mosquitoes, as removing standing water from outdoor containers (49.5%), mowing the lawn (35.3%), and applying larvicides to drains or containers (18.7%) (Table 5).

**Table 5:** Methods usually used to protect against mosquitoes (%; *n* = 1,001).

Practices	%
I use skin-applied repellents	62.6
I use environmental repellents (mosquito coils, electric diffusers, candles, etc.)	60.1
I use mosquito nets	53.5
I remove water from containers/keep them upside down/make sure I do not have any	49.5
I mow the lawn	35.3
I use air conditioning or fans	31.6
I try to stay indoors in the late afternoon or evening	22.2
I put larvicides in drains/containers (treatment against larvae)	18.7
I wear appropriate clothing (protective, light-colored, etc.)	17.2
I use electric lamps outside the house	11.7
I carry out adulticide treatments (against adult mosquitoes)	11.6
I cover large containers/gutters with nets or lids	10
I contact a pest control company	3.3
Other	3.3

The local entities most frequently identified as key participants in the fight against mosquitoes were municipal authorities (63%), followed by land reclamation authorities (58.8%), and local health authorities (52.6%). Additionally, 40.8% of respondents identified citizens as important stakeholders in mosquito control efforts.

### 3.5 Factors influencing risk perceptions

Using the independent *t*-test, the RP index proved higher in females compared to males (*p* = 0.002) and among respondents who reported having recently read or heard news about mosquitoes in the newspaper or on television compared to those who had not (*p* < 0.001). ANOVA analysis revealed that the RP index varied by educational level (*p* = 0.001). According to Gabriel’s *post-hoc* correction, respondents with a primary/middle school (mean RP index = 3.753) or high school diploma (mean RP index = 3.630) showed higher risk perception than those with a university degree (mean RP index = 3.244, *p* = 0.001 and *p* = 0.007, respectively). Regarding employment status, students reported lower risk perception (mean RP index = 2.859) compared to the employed (mean RP index = 3.529, *p* = 0.004), unemployed (mean RP index = 3.795, *p* = 0.009), homemakers (average RP index 3.905, *p* = 0.001), and retirees (mean RP index = 3.633, *p* = 0.002).

No significant relationships were observed between the RP index and respondents’ age (*p* = 0.333), the presence of children in their household (*p* = 0.066), the setting (urban, suburban, rural) in which they lived (*p* = 0.989), and whether they had traveled to tropical countries (*p* = 0.940).

Respondents’ knowledge of mosquito ecology and biology was inversely correlated with the RP index, indicating that those with a higher level of knowledge expressed a lower degree of risk perception (*p* = 0.001).

The RP index was positively correlated with: (1) the “protective behaviors” variable, indicating that higher risk perception levels were associated with a higher tendency to adopt protective behaviors (*p* = 0.001), and (2) descriptive social norms, i.e., the perception that other people living in the area considered mosquitoes to be very annoying (*p* < 0.001) and to be a serious threat to the community (*p* < 0.001).

## 4 Discussion

This study aimed to investigate citizens’ attitudes, knowledge, and preventive behaviors in relation to mosquitoes and MBDs in two regions of northeastern Italy. The findings revealed several important insights into how individuals assessed and responded to the perceived threat of mosquitoes, both as a nuisance and as a potential vector of disease.

### 4.1 Attitudes and perceptions

Respondents perceived a high presence of mosquitoes in their areas of residence, with nearly half observing an increase over the past 2–3 years. However, these perceptions are not supported by entomological surveillance data for WNV vectors, which show that mosquito populations have remained relatively stable over the past 10 years (32). In the study, perceived abundance did not automatically translate into heightened perceptions of health risk. While mosquitoes were widely considered a nuisance, the perceived threat to personal health remained moderate. This discrepancy between nuisance perception and risk perception has also been observed in other European settings and may reflect low levels of perceived vulnerability (29, 33, 34). Furthermore, respondents expressed significantly lower concern when assessing risk at the local compared to the national level. This suggests that respondents underestimated the risks to themselves and their local environment, which may be linked to psychological factors such as perceived invulnerability and perceived control over own surroundings (35, 36).

### 4.2 Knowledge

Knowledge levels varied across domains. Respondents demonstrated good knowledge about environmental factors driving mosquito proliferation, including climate change. They also showed strong awareness of mosquito breeding sites—particularly water-filled containers and natural stagnant water, consistent with findings from other studies where standing water was widely acknowledged to be a potential breeding site (34, 37). However, some misconceptions did emerge about mosquito biology, since a large proportion of respondents incorrectly believed that mosquitoes feed exclusively on blood or that both males and females bite.

The majority of respondents correctly identified the presence of both native and invasive mosquitoes, although nearly one in three expressed uncertainty. Good knowledge about the presence of invasive species is due to the establishment of *Aedes albopictus* (the Asian tiger mosquito) in Italy for over 30 years (38). However, the more recent introduction of other non-native species is still ongoing and the introduction of additional invasive species (*Aedes koreicus* and *Ae. japonicus*) presents new challenges for mosquito management and control.

Respondents were aware that mosquitoes can transmit pathogens causing disease and that different species can transmit different pathogens. This becomes even more significant in view of their limited knowledge of the MBDs present in their own area of residence. The best-known disease was WND, which has become endemic in the two regions considered (9, 39). Conversely, public perception of malaria did not correspond to scientific evidence: one in five respondents believed it was still present in their area in recent years and the same percentage of respondents did not know whether or not it was present. Although malaria historically affected some areas of the Po River Valley, Italy has officially been declared malaria free by the World Health Organization (WHO) since 1970. Nonetheless, mosquitoes of the *Anopheles maculipennis* complex—vectors of the *Plasmodium* parasite—are still present in the area (7). Several European studies have shown similar gaps in MDB knowledge, likely reflecting the sporadic nature of local transmission and the predominance of outbreaks linked to imported cases (40, 41). A study conducted in central Italy comparing the KAP of an Italian sample with two Indian communities showed that while people from endemic areas were more concerned and more knowledgeable about MBDs, they perceived mosquitoes less as a nuisance (42).

### 4.3 Protection and control practices

Encouragingly, respondents reported engaging in a broad set of PPBs, particularly the use of repellents and physical barriers, such as mosquito nets. Respondents also practiced environmental prevention measures, including the removal of standing water. One in five respondents avoided going outdoors in the afternoon or evening to reduce mosquito bites. This is in line with other studies where people reported changing their daily activities to avoid bites (19).

A growing body of evidence has demonstrated that the effectiveness of mosquito control interventions and the prevention of MBDs is strongly influenced by the adoption of appropriate PPBs among the population. Several studies (34, 43) have identified individual awareness of health risks as a key determinant in promoting mosquito control practices and encouraging the adoption of PPBs. Therefore, understanding population attitudes toward mosquitoes and MBD becomes key to the design of effective, targeted health communication strategies.

Given the absence of vaccines for most MBD, primary prevention relies on vector control strategies and the adoption of PPBs (44). Notably, empirical studies have estimated that regular application of PPBs can reduce the risk of WNV infection by approximately 50%, making them the most effective form of personal prophylaxis (45).

Municipal authorities and health services were widely seen as responsible for mosquito control, while only 40.8% identified citizens themselves as key stakeholders. This reveals a gap in public understanding of integrated vector management, where community engagement is considered a cornerstone of effective, sustainable mosquito control. On the other hand, the way citizens are involved and the effectiveness of interventions is a pivotal issue (33, 46). Hence, intervention planning requires not only institutional leadership but also systematic evaluation of citizen involvement in terms of the cost-effectiveness of control strategies.

### 4.4 Factors influencing risk perception

The study found that individuals with higher risk perception scores (RP index) were more likely to engage in PPBs, confirming the central role of perceived threat in shaping health-related actions. This result is consistent with psychological frameworks that posit perceived susceptibility and severity as key predictors of protective behavior (47, 48). Importantly, PPBs are not determined solely by risk perception; they are also associated with disease-related knowledge and sociodemographic variables (19, 20). A systematic review on KAP and their determinants found that concern about WNV circulation appeared to be a predictor of all examined preventive behaviors (49). The correlation between risk perception and behavioral practices is consolidated by other studies on different MBDs (34, 43).

With regard to sociodemographic variables, the survey revealed that gender, educational attainment, and occupational status significantly influenced perceived risk. Women reported higher risk perception than men, a finding consistent with existing literature, suggesting that women tend to express greater concern toward health threats (27, 29, 50). Respondents with lower educational levels expressed higher perceived risk compared to those with tertiary education. While this may reflect unequal access to scientific information, it could also suggest a heightened sense of vulnerability among individuals with less formal education (51, 52). Furthermore, students reported significantly lower risk perceptions compared to employed or retired respondents. This could reflect both lower feelings of vulnerability or less attention to vector-borne disease threats in public communication interventions targeting young people. A study conducted in Australia found that younger people exhibited low risk perception and limited knowledge of MBDs, identifying them as an important target for tailored risk communication (29).

Conversely, risk perception was negatively associated with level of scientific knowledge about mosquito biology and ecology. More knowledgeable respondents tended to report lower levels of perceived risk. This pattern may reflect informed individuals’ greater ability to contextualize the actual health threat posed by mosquitoes considering that the probability of infection is still relatively rare in Italy. Another possible explanation is that knowledge can foster protective behaviors, which increase feelings of control and safety, thereby reducing risk perception (53). This finding is consistent with other studies that showed that higher levels of knowledge can sometimes lead to more rational, and thus lower, risk appraisal (54, 55).

The study also revealed that descriptive social norms were positively associated with perceived risk. This aligns with Ojala and Lidskog (50), who found that individuals who perceived their community to be highly disturbed by mosquitoes were more likely to develop heightened risk perceptions, corroborating broader evidence on the impact of descriptive norms on risk appraisal (56, 57). Moreover, results showed that exposure to media reports influenced risk perception. Annual outbreaks of diseases such as WNV and dengue in certain areas, often amplified by national media coverage, can increase perceived risk even among individuals with no direct personal experience. According to the theory of social amplification of risk, exposure to mosquito-related news and shared information in informal networks was associated with increased concern, suggesting that media coverage and social relationships remain powerful tools for amplifying risk perception and fostering public engagement (58). This effect can become even stronger when the issue has a local dimension, as in the case of mosquito-related risks, in which case the community shares information, management initiatives, and interventions.

The results of this study reinforce the idea that risk perception is shaped by social and emotional cues, including media salience and perceived social attitudes, which may serve as important triggers for action (21, 26, 29).

When interpreting the results, it is important to contextualize the period in which the survey took place and recognize the possible impact of the COVID-19 period on the public perception of risks and especially of MBDs. In the spring of 2020, the rapid spread of COVID-19 in Italy led to profound changes in individual behaviors and required the adoption of extensive personal and collective protective measures. How this global event—with its substantial health, economic, and social consequences—affected vector-borne diseases has been the focus of several studies. These analyses highlight a contraction in resources dedicated to prevention and control activities, potentially resulting in underdiagnosis and underestimation of cases on the one hand, and a possible increase in vector populations on the other (59). With regard to public awareness, the interruption of information campaigns and community-engagement activities may have reduced public attention to MBDs and a downgrading of their perceived risk relative to the pandemic emergency (60). Moreover, media coverage of mosquito-borne diseases may also have been influenced by the predominance of COVID-19 as the major public health concern during that period. It would be interesting to further investigate how COVID 19 may have affected the relationship between risk perception, protective behaviors and other factors such as social norms in the MBDs domain.

The results of this study showed a perceived increase in mosquitoes presence that may be linked to an actual rise in their numbers due to the reduction of control interventions in urban areas during the pandemic, but it could also be the result of heightened threat perception caused by the pandemic itself. However, these hypothesis cannot be confirmed by entomological data in the considered territory, at least for *Aedes albopictus* (the main urban species in Italy), as it was not possible to carry out monitoring in 2020 due to restrictions imposed by COVID-19. On the other hand, entomological surveillance for WNV in 2020 (conducted regularly) did not report a significant difference in the number of *Cx. pipiens* captured.

### 4.5 Policy implications and recommendations

Understanding people’s perceptions and opinions about mosquitoes, as well as their attitudes toward these insects is an essential part in the design of risk communication strategies and in planning citizen engagement in preventive activities. These efforts should build awareness that everyone can contribute to protecting public health. By adopting correct PPBs, citizens play an important role in mosquito density control and, consequently, in the incidence of MBDs. To be effective, strategies to mitigate health risks associated with mosquitoes must therefore adopt an interdisciplinary, multi-stakeholder approach that combines epidemiological surveillance, education, communication, and community mobilization. Based on the study findings, several recommendations can be proposed to improve mosquito control efforts and public health communication strategies.

Enhanced public awareness campaigns:

- Information campaigns should aim to correct misconceptions about mosquito biology and increase awareness of MBDs and transmission. Communication strategies should aim not only to raise awareness about mosquitoes as a nuisance but also to bridge the gap between discomfort and actual health risk. Alongside PPBs, more sustainable environmental control measures should also be promoted. Messages must be scientifically accurate, tailored to the local setting, and accessible across different educational levels. According to the study results, interventions should target specific population subgroups (e.g., men, individuals with higher education, students) who may underestimate the risk.

Community engagement in vector control:

- Stepping up community-based strategies and reinforcing collective responsibility (e.g., via municipalities and local health authorities) could promote more consistent, sustainable mosquito control efforts aligned with the Integrated Vector Management (IVM) strategy recommended by WHO. This could encompass citizen science initiatives, neighborhood surveillance, or education and training programs on simple preventive actions combining PPBs with community-wide environmental management.

Leverage media and social norms:

- Since study results show that both media exposure and descriptive social norms are associated with risk perception, health authorities should strategically use media and social influence frameworks to enhance perceived relevance without inducing unnecessary alarm. Media should convey a balanced, scientifically accurate assessment of mosquito-related threats. At local level, health authorities should foster a widespread culture of prevention in the community to raise risk awareness among citizens.

Support local authorities in the promotion of sustainable control measures:

- Efforts by municipal and regional health authorities should be supported through appropriate resource allocation, technical guidance, and coordination platforms to ensure alignment between public services and citizen initiatives. Larval source management should be promoted as a long-term mosquito control strategy in both public and private settings. To track the effectiveness of public health interventions, continuous surveillance of KAP should be implemented, ideally integrated with entomological and epidemiological data. This is especially important in temperate regions which are experiencing ecological change.

## 5 Limitations

While this study offers valuable insights into mosquito-related perceptions and behaviors in northeastern Italy, several limitations should be acknowledged. While improving demographic coverage, the mixed-mode data collection strategy may have introduced mode-related response biases, potentially overrepresenting individuals with higher digital literacy or pre-existing interest in the topic (61, 62). Moreover, mixed-mode surveys can be subject to the mode measurement effect, which is related to the way in which the administration method itself influences the question-answer process. To adjust this effect, the survey was carefully conducted using a unified mode design (63), for example, using questions and response formats limited to simple options that are easily understandable in the telephone mode. This sought to avoid differences in design, questionnaire, and implementation, in order to achieve measurement equivalence (64) and obtain the same responses in both modes. The pre-test, conducted both through self-completion and with the support of an interviewer, simulating a telephone interview, aimed to verify question clarity and avoid ambiguous interpretation across the two response modes. Additionally, reliance on self-reported data may have given rise to social desirability bias, particularly regarding preventive practices and perceived responsibility. However, the mixed mode is considered a method that helps reduce this bias, which is usually more evident in interviewer-led telephone interviews (61). Finally, regional generalizability is limited; future studies should aim to replicate the findings across diverse geographical and cultural contexts within Italy and beyond.

## Data Availability

The raw data supporting the conclusions of this article will be made available by the authors, without undue reservation.

## Footnotes

1
http://www.opinioni.net
